# Supplementary material for: Computing mathematical functions with chemical reactions via stochastic logic
Source: PLoS One. 2023 May 8;18(5):e0281574. doi: 10.1371/journal.pone.0281574 (PMC10166555; doi:10.1371/journal.pone.0281574)
Supplement: S1 File — We calculate the CRN for polynomial approximations of various functions such as ArcTan, Exponential, Bessel, and Sinc. (PDF) [file pone.0281574.s001.pdf]

Supplementary Information S1

Examples of CRNs for polynomial approximations of nonlinear functions.

ArcTan Function

Here we show an example of $\arctan(x)$. It can be approximated as:

$$\arctan(x) \approx x - \frac{1}{3}x^3 = x(1 - \frac{1}{3}x^2).$$

We assign the stochastic variables $x_1 = x, x_2 = \frac{1}{3}, x_3 = x_4 = x$. Then the stochastic logic function is:

$$\mathrm{AND}(x_1, \mathrm{NAND}(x_2, \mathrm{AND}(x_3, x_4))).$$

According to the truth table, the corresponding CRN is:

$$\mathbf{X}_{1,0} + \mathbf{X}_{2,0} + \mathbf{X}_{3,0} + \mathbf{X}_{4,0} \to \mathbf{Y}_0$$
$$\mathbf{X}_{1,0} + \mathbf{X}_{2,0} + \mathbf{X}_{3,0} + \mathbf{X}_{4,1} \to \mathbf{Y}_0$$
$$\mathbf{X}_{1,0} + \mathbf{X}_{2,0} + \mathbf{X}_{3,1} + \mathbf{X}_{4,0} \to \mathbf{Y}_0$$
$$\mathbf{X}_{1,0} + \mathbf{X}_{2,0} + \mathbf{X}_{3,1} + \mathbf{X}_{4,1} \to \mathbf{Y}_0$$
$$\mathbf{X}_{1,0} + \mathbf{X}_{2,1} + \mathbf{X}_{3,0} + \mathbf{X}_{4,0} \to \mathbf{Y}_0$$
$$\mathbf{X}_{1,0} + \mathbf{X}_{2,1} + \mathbf{X}_{3,0} + \mathbf{X}_{4,1} \to \mathbf{Y}_0$$
$$\mathbf{X}_{1,0} + \mathbf{X}_{2,1} + \mathbf{X}_{3,1} + \mathbf{X}_{4,0} \to \mathbf{Y}_0$$
$$\mathbf{X}_{1,0} + \mathbf{X}_{2,1} + \mathbf{X}_{3,1} + \mathbf{X}_{4,1} \to \mathbf{Y}_0$$
$$\mathbf{X}_{1,1} + \mathbf{X}_{2,0} + \mathbf{X}_{3,0} + \mathbf{X}_{4,0} \to \mathbf{Y}_1$$
$$\mathbf{X}_{1,1} + \mathbf{X}_{2,0} + \mathbf{X}_{3,0} + \mathbf{X}_{4,1} \to \mathbf{Y}_1$$
$$\mathbf{X}_{1,1} + \mathbf{X}_{2,0} + \mathbf{X}_{3,1} + \mathbf{X}_{4,0} \to \mathbf{Y}_1$$
$$\mathbf{X}_{1,1} + \mathbf{X}_{2,0} + \mathbf{X}_{3,1} + \mathbf{X}_{4,1} \to \mathbf{Y}_1$$
$$\mathbf{X}_{1,1} + \mathbf{X}_{2,1} + \mathbf{X}_{3,0} + \mathbf{X}_{4,0} \to \mathbf{Y}_1$$
$$\mathbf{X}_{1,1} + \mathbf{X}_{2,1} + \mathbf{X}_{3,0} + \mathbf{X}_{4,1} \to \mathbf{Y}_1$$
$$\mathbf{X}_{1,1} + \mathbf{X}_{2,1} + \mathbf{X}_{3,1} + \mathbf{X}_{4,0} \to \mathbf{Y}_1$$
$$\mathbf{X}_{1,1} + \mathbf{X}_{2,1} + \mathbf{X}_{3,1} + \mathbf{X}_{4,1} \to \mathbf{Y}_0$$

Exponential Function
Here we show an example of $\exp(-x)$. Its approximation is:

$$\exp(-x) \approx 1 - x + \frac{1}{2}x^2 - \frac{1}{6}x^3 = 1 - x(1 - \frac{1}{2}x(1 - \frac{1}{3}x))$$

We assign the stochastic variables $x_1 = x, x_2 = \frac{1}{2}, x_3 = x, x_4 = \frac{1}{3}, x_5 = x$. Then the stochastic logic function is:

$$\mathrm{NAND}(x_1, \mathrm{NAND}(x_2, \mathrm{AND}(x_3, \mathrm{NAND}(x_4, x_5)))).$$

According to the truth table, the corresponding CRN is:

$$\begin{aligned}
&\mathbf{X}_{1,0} + \mathbf{X}_{2,0} + \mathbf{X}_{3,0} + \mathbf{X}_{4,0} + \mathbf{X}_{5,0} \rightarrow \mathbf{Y}_1\\
&\mathbf{X}_{1,0} + \mathbf{X}_{2,0} + \mathbf{X}_{3,0} + \mathbf{X}_{4,0} + \mathbf{X}_{5,1} \rightarrow \mathbf{Y}_1\\
&\mathbf{X}_{1,0} + \mathbf{X}_{2,0} + \mathbf{X}_{3,0} + \mathbf{X}_{4,1} + \mathbf{X}_{5,0} \rightarrow \mathbf{Y}_1\\
&\mathbf{X}_{1,0} + \mathbf{X}_{2,0} + \mathbf{X}_{3,0} + \mathbf{X}_{4,1} + \mathbf{X}_{5,1} \rightarrow \mathbf{Y}_1\\
&\mathbf{X}_{1,0} + \mathbf{X}_{2,0} + \mathbf{X}_{3,1} + \mathbf{X}_{4,0} + \mathbf{X}_{5,0} \rightarrow \mathbf{Y}_1\\
&\mathbf{X}_{1,0} + \mathbf{X}_{2,0} + \mathbf{X}_{3,1} + \mathbf{X}_{4,0} + \mathbf{X}_{5,1} \rightarrow \mathbf{Y}_1\\
&\mathbf{X}_{1,0} + \mathbf{X}_{2,0} + \mathbf{X}_{3,1} + \mathbf{X}_{4,1} + \mathbf{X}_{5,0} \rightarrow \mathbf{Y}_1\\
&\mathbf{X}_{1,0} + \mathbf{X}_{2,0} + \mathbf{X}_{3,1} + \mathbf{X}_{4,1} + \mathbf{X}_{5,1} \rightarrow \mathbf{Y}_1\\
&\mathbf{X}_{1,0} + \mathbf{X}_{2,1} + \mathbf{X}_{3,0} + \mathbf{X}_{4,0} + \mathbf{X}_{5,0} \rightarrow \mathbf{Y}_1\\
&\mathbf{X}_{1,0} + \mathbf{X}_{2,1} + \mathbf{X}_{3,0} + \mathbf{X}_{4,0} + \mathbf{X}_{5,1} \rightarrow \mathbf{Y}_1\\
&\mathbf{X}_{1,0} + \mathbf{X}_{2,1} + \mathbf{X}_{3,0} + \mathbf{X}_{4,1} + \mathbf{X}_{5,0} \rightarrow \mathbf{Y}_1\\
&\mathbf{X}_{1,0} + \mathbf{X}_{2,1} + \mathbf{X}_{3,0} + \mathbf{X}_{4,1} + \mathbf{X}_{5,1} \rightarrow \mathbf{Y}_1\\
&\mathbf{X}_{1,0} + \mathbf{X}_{2,1} + \mathbf{X}_{3,1} + \mathbf{X}_{4,0} + \mathbf{X}_{5,0} \rightarrow \mathbf{Y}_1\\
&\mathbf{X}_{1,0} + \mathbf{X}_{2,1} + \mathbf{X}_{3,1} + \mathbf{X}_{4,0} + \mathbf{X}_{5,1} \rightarrow \mathbf{Y}_1\\
&\mathbf{X}_{1,0} + \mathbf{X}_{2,1} + \mathbf{X}_{3,1} + \mathbf{X}_{4,1} + \mathbf{X}_{5,0} \rightarrow \mathbf{Y}_1\\
&\mathbf{X}_{1,0} + \mathbf{X}_{2,1} + \mathbf{X}_{3,1} + \mathbf{X}_{4,1} + \mathbf{X}_{5,1} \rightarrow \mathbf{Y}_1\\
&\mathbf{X}_{1,1} + \mathbf{X}_{2,0} + \mathbf{X}_{3,0} + \mathbf{X}_{4,0} + \mathbf{X}_{5,0} \rightarrow \mathbf{Y}_0\\
&\mathbf{X}_{1,1} + \mathbf{X}_{2,0} + \mathbf{X}_{3,0} + \mathbf{X}_{4,0} + \mathbf{X}_{5,1} \rightarrow \mathbf{Y}_0\\
&\mathbf{X}_{1,1} + \mathbf{X}_{2,0} + \mathbf{X}_{3,0} + \mathbf{X}_{4,1} + \mathbf{X}_{5,0} \rightarrow \mathbf{Y}_0\\
&\mathbf{X}_{1,1} + \mathbf{X}_{2,0} + \mathbf{X}_{3,0} + \mathbf{X}_{4,1} + \mathbf{X}_{5,1} \rightarrow \mathbf{Y}_0\\
&\mathbf{X}_{1,1} + \mathbf{X}_{2,0} + \mathbf{X}_{3,1} + \mathbf{X}_{4,0} + \mathbf{X}_{5,0} \rightarrow \mathbf{Y}_0\\
&\mathbf{X}_{1,1} + \mathbf{X}_{2,0} + \mathbf{X}_{3,1} + \mathbf{X}_{4,0} + \mathbf{X}_{5,1} \rightarrow \mathbf{Y}_0\\
&\mathbf{X}_{1,1} + \mathbf{X}_{2,0} + \mathbf{X}_{3,1} + \mathbf{X}_{4,1} + \mathbf{X}_{5,0} \rightarrow \mathbf{Y}_0\\
&\mathbf{X}_{1,1} + \mathbf{X}_{2,0} + \mathbf{X}_{3,1} + \mathbf{X}_{4,1} + \mathbf{X}_{5,1} \rightarrow \mathbf{Y}_0\\
&\mathbf{X}_{1,1} + \mathbf{X}_{2,1} + \mathbf{X}_{3,0} + \mathbf{X}_{4,0} + \mathbf{X}_{5,0} \rightarrow \mathbf{Y}_0\\
&\mathbf{X}_{1,1} + \mathbf{X}_{2,1} + \mathbf{X}_{3,0} + \mathbf{X}_{4,0} + \mathbf{X}_{5,1} \rightarrow \mathbf{Y}_0\\
&\mathbf{X}_{1,1} + \mathbf{X}_{2,1} + \mathbf{X}_{3,0} + \mathbf{X}_{4,1} + \mathbf{X}_{5,0} \rightarrow \mathbf{Y}_0\\
&\mathbf{X}_{1,1} + \mathbf{X}_{2,1} + \mathbf{X}_{3,0} + \mathbf{X}_{4,1} + \mathbf{X}_{5,1} \rightarrow \mathbf{Y}_0\\
&\mathbf{X}_{1,1} + \mathbf{X}_{2,1} + \mathbf{X}_{3,1} + \mathbf{X}_{4,0} + \mathbf{X}_{5,0} \rightarrow \mathbf{Y}_1\\
&\mathbf{X}_{1,1} + \mathbf{X}_{2,1} + \mathbf{X}_{3,1} + \mathbf{X}_{4,0} + \mathbf{X}_{5,1} \rightarrow \mathbf{Y}_1\\
&\mathbf{X}_{1,1} + \mathbf{X}_{2,1} + \mathbf{X}_{3,1} + \mathbf{X}_{4,1} + \mathbf{X}_{5,0} \rightarrow \mathbf{Y}_1\\
&\mathbf{X}_{1,1} + \mathbf{X}_{2,1} + \mathbf{X}_{3,1} + \mathbf{X}_{4,1} + \mathbf{X}_{5,1} \rightarrow \mathbf{Y}_0
\end{aligned}$$

Bessel Function

Here we show an example of the Bessel function of the first kind with parameter $\alpha = 1$. Its approximation is:

$$J_1(x) \approx \frac{1}{2}x - \frac{1}{16}x^3 = \frac{1}{2}x(1 - \frac{1}{8}x^2).$$

We assign the stochastic variables $x_1 = \frac{1}{2}, x_2 = x, x_3 = \frac{1}{8}, x_4 = x_5 = x$. Then the stochastic logic function is:

$$\text{AND}(x_1, \text{AND}(x_2, \text{NAND}(x_3, \text{AND}(x_4, x_5)))).$$

According to the truth table, the corresponding CRN is:

$$\mathbf{X}_{1,0} + \mathbf{X}_{2,0} + \mathbf{X}_{3,0} + \mathbf{X}_{4,0} + \mathbf{X}_{5,0} \rightarrow \mathbf{Y}_0$$
$$\mathbf{X}_{1,0} + \mathbf{X}_{2,0} + \mathbf{X}_{3,0} + \mathbf{X}_{4,0} + \mathbf{X}_{5,1} \rightarrow \mathbf{Y}_0$$
$$\mathbf{X}_{1,0} + \mathbf{X}_{2,0} + \mathbf{X}_{3,0} + \mathbf{X}_{4,1} + \mathbf{X}_{5,0} \rightarrow \mathbf{Y}_0$$
$$\mathbf{X}_{1,0} + \mathbf{X}_{2,0} + \mathbf{X}_{3,0} + \mathbf{X}_{4,1} + \mathbf{X}_{5,1} \rightarrow \mathbf{Y}_0$$
$$\mathbf{X}_{1,0} + \mathbf{X}_{2,0} + \mathbf{X}_{3,1} + \mathbf{X}_{4,0} + \mathbf{X}_{5,0} \rightarrow \mathbf{Y}_0$$
$$\mathbf{X}_{1,0} + \mathbf{X}_{2,0} + \mathbf{X}_{3,1} + \mathbf{X}_{4,0} + \mathbf{X}_{5,1} \rightarrow \mathbf{Y}_0$$
$$\mathbf{X}_{1,0} + \mathbf{X}_{2,0} + \mathbf{X}_{3,1} + \mathbf{X}_{4,1} + \mathbf{X}_{5,0} \rightarrow \mathbf{Y}_0$$
$$\mathbf{X}_{1,0} + \mathbf{X}_{2,0} + \mathbf{X}_{3,1} + \mathbf{X}_{4,1} + \mathbf{X}_{5,1} \rightarrow \mathbf{Y}_0$$
$$\mathbf{X}_{1,0} + \mathbf{X}_{2,1} + \mathbf{X}_{3,0} + \mathbf{X}_{4,0} + \mathbf{X}_{5,0} \rightarrow \mathbf{Y}_0$$
$$\mathbf{X}_{1,0} + \mathbf{X}_{2,1} + \mathbf{X}_{3,0} + \mathbf{X}_{4,0} + \mathbf{X}_{5,1} \rightarrow \mathbf{Y}_0$$
$$\mathbf{X}_{1,0} + \mathbf{X}_{2,1} + \mathbf{X}_{3,0} + \mathbf{X}_{4,1} + \mathbf{X}_{5,0} \rightarrow \mathbf{Y}_0$$
$$\mathbf{X}_{1,0} + \mathbf{X}_{2,1} + \mathbf{X}_{3,0} + \mathbf{X}_{4,1} + \mathbf{X}_{5,1} \rightarrow \mathbf{Y}_0$$
$$\mathbf{X}_{1,0} + \mathbf{X}_{2,1} + \mathbf{X}_{3,1} + \mathbf{X}_{4,0} + \mathbf{X}_{5,0} \rightarrow \mathbf{Y}_0$$
$$\mathbf{X}_{1,0} + \mathbf{X}_{2,1} + \mathbf{X}_{3,1} + \mathbf{X}_{4,0} + \mathbf{X}_{5,1} \rightarrow \mathbf{Y}_0$$
$$\mathbf{X}_{1,0} + \mathbf{X}_{2,1} + \mathbf{X}_{3,1} + \mathbf{X}_{4,1} + \mathbf{X}_{5,0} \rightarrow \mathbf{Y}_0$$
$$\mathbf{X}_{1,0} + \mathbf{X}_{2,1} + \mathbf{X}_{3,1} + \mathbf{X}_{4,1} + \mathbf{X}_{5,1} \rightarrow \mathbf{Y}_0$$
$$\mathbf{X}_{1,1} + \mathbf{X}_{2,0} + \mathbf{X}_{3,0} + \mathbf{X}_{4,0} + \mathbf{X}_{5,0} \rightarrow \mathbf{Y}_0$$
$$\mathbf{X}_{1,1} + \mathbf{X}_{2,0} + \mathbf{X}_{3,0} + \mathbf{X}_{4,0} + \mathbf{X}_{5,1} \rightarrow \mathbf{Y}_0$$
$$\mathbf{X}_{1,1} + \mathbf{X}_{2,0} + \mathbf{X}_{3,0} + \mathbf{X}_{4,1} + \mathbf{X}_{5,0} \rightarrow \mathbf{Y}_0$$
$$\mathbf{X}_{1,1} + \mathbf{X}_{2,0} + \mathbf{X}_{3,0} + \mathbf{X}_{4,1} + \mathbf{X}_{5,1} \rightarrow \mathbf{Y}_0$$
$$\mathbf{X}_{1,1} + \mathbf{X}_{2,0} + \mathbf{X}_{3,1} + \mathbf{X}_{4,0} + \mathbf{X}_{5,0} \rightarrow \mathbf{Y}_0$$
$$\mathbf{X}_{1,1} + \mathbf{X}_{2,0} + \mathbf{X}_{3,1} + \mathbf{X}_{4,0} + \mathbf{X}_{5,1} \rightarrow \mathbf{Y}_0$$
$$\mathbf{X}_{1,1} + \mathbf{X}_{2,0} + \mathbf{X}_{3,1} + \mathbf{X}_{4,1} + \mathbf{X}_{5,0} \rightarrow \mathbf{Y}_0$$
$$\mathbf{X}_{1,1} + \mathbf{X}_{2,0} + \mathbf{X}_{3,1} + \mathbf{X}_{4,1} + \mathbf{X}_{5,1} \rightarrow \mathbf{Y}_0$$
$$\mathbf{X}_{1,1} + \mathbf{X}_{2,1} + \mathbf{X}_{3,0} + \mathbf{X}_{4,0} + \mathbf{X}_{5,0} \rightarrow \mathbf{Y}_1$$
$$\mathbf{X}_{1,1} + \mathbf{X}_{2,1} + \mathbf{X}_{3,0} + \mathbf{X}_{4,0} + \mathbf{X}_{5,1} \rightarrow \mathbf{Y}_1$$

$$\mathbf{X}_{1,1} + \mathbf{X}_{2,1} + \mathbf{X}_{3,0} + \mathbf{X}_{4,1} + \mathbf{X}_{5,0} \rightarrow \mathbf{Y}_1$$
$$\mathbf{X}_{1,1} + \mathbf{X}_{2,1} + \mathbf{X}_{3,0} + \mathbf{X}_{4,1} + \mathbf{X}_{5,1} \rightarrow \mathbf{Y}_1$$
$$\mathbf{X}_{1,1} + \mathbf{X}_{2,1} + \mathbf{X}_{3,1} + \mathbf{X}_{4,0} + \mathbf{X}_{5,0} \rightarrow \mathbf{Y}_1$$
$$\mathbf{X}_{1,1} + \mathbf{X}_{2,1} + \mathbf{X}_{3,1} + \mathbf{X}_{4,0} + \mathbf{X}_{5,1} \rightarrow \mathbf{Y}_1$$
$$\mathbf{X}_{1,1} + \mathbf{X}_{2,1} + \mathbf{X}_{3,1} + \mathbf{X}_{4,1} + \mathbf{X}_{5,0} \rightarrow \mathbf{Y}_1$$
$$\mathbf{X}_{1,1} + \mathbf{X}_{2,1} + \mathbf{X}_{3,1} + \mathbf{X}_{4,1} + \mathbf{X}_{5,1} \rightarrow \mathbf{Y}_0$$

Sinc Function
The mathematical expression of the sinc function is:

$$\mathrm{sinc}(x) = \frac{\sin(x)}{x}.$$

Its approximation is:

$$\mathrm{sinc}(x) \approx 1 - \frac{1}{6}x^2 + \frac{1}{120}x^4 = 1 - \frac{1}{6}x^2(1 - \frac{1}{20}x^2)$$

We assign the stochastic variables $x_1 = x_2 = x, x_3 = \frac{1}{6}, x_4 = \frac{1}{20}, x_5 = x_6 = x$. Then the stochastic logic function is:

$$\mathrm{NAND}(\mathrm{AND}(x_1, x_2), \mathrm{AND}(x_3, \mathrm{NAND}(x_4, \mathrm{AND}(x_5, x_6)))).$$

According to the truth table, the corresponding CRN is:

$$\mathbf{X}_{1,0} + \mathbf{X}_{2,0} + \mathbf{X}_{3,0} + \mathbf{X}_{4,0} + \mathbf{X}_{5,0} + \mathbf{X}_{6,0} \to \mathbf{Y}_1$$
$$\mathbf{X}_{1,0} + \mathbf{X}_{2,0} + \mathbf{X}_{3,0} + \mathbf{X}_{4,0} + \mathbf{X}_{5,0} + \mathbf{X}_{6,1} \to \mathbf{Y}_1$$
$$\mathbf{X}_{1,0} + \mathbf{X}_{2,0} + \mathbf{X}_{3,0} + \mathbf{X}_{4,0} + \mathbf{X}_{5,1} + \mathbf{X}_{6,0} \to \mathbf{Y}_1$$
$$\mathbf{X}_{1,0} + \mathbf{X}_{2,0} + \mathbf{X}_{3,0} + \mathbf{X}_{4,0} + \mathbf{X}_{5,1} + \mathbf{X}_{6,1} \to \mathbf{Y}_1$$
$$\mathbf{X}_{1,0} + \mathbf{X}_{2,0} + \mathbf{X}_{3,0} + \mathbf{X}_{4,1} + \mathbf{X}_{5,0} + \mathbf{X}_{6,0} \to \mathbf{Y}_1$$
$$\mathbf{X}_{1,0} + \mathbf{X}_{2,0} + \mathbf{X}_{3,0} + \mathbf{X}_{4,1} + \mathbf{X}_{5,0} + \mathbf{X}_{6,1} \to \mathbf{Y}_1$$
$$\mathbf{X}_{1,0} + \mathbf{X}_{2,0} + \mathbf{X}_{3,0} + \mathbf{X}_{4,1} + \mathbf{X}_{5,1} + \mathbf{X}_{6,0} \to \mathbf{Y}_1$$
$$\mathbf{X}_{1,0} + \mathbf{X}_{2,0} + \mathbf{X}_{3,0} + \mathbf{X}_{4,1} + \mathbf{X}_{5,1} + \mathbf{X}_{6,1} \to \mathbf{Y}_1$$
$$\mathbf{X}_{1,0} + \mathbf{X}_{2,0} + \mathbf{X}_{3,1} + \mathbf{X}_{4,0} + \mathbf{X}_{5,0} + \mathbf{X}_{6,0} \to \mathbf{Y}_1$$
$$\mathbf{X}_{1,0} + \mathbf{X}_{2,0} + \mathbf{X}_{3,1} + \mathbf{X}_{4,0} + \mathbf{X}_{5,0} + \mathbf{X}_{6,1} \to \mathbf{Y}_1$$
$$\mathbf{X}_{1,0} + \mathbf{X}_{2,0} + \mathbf{X}_{3,1} + \mathbf{X}_{4,0} + \mathbf{X}_{5,1} + \mathbf{X}_{6,0} \to \mathbf{Y}_1$$
$$\mathbf{X}_{1,0} + \mathbf{X}_{2,0} + \mathbf{X}_{3,1} + \mathbf{X}_{4,0} + \mathbf{X}_{5,1} + \mathbf{X}_{6,1} \to \mathbf{Y}_1$$
$$\mathbf{X}_{1,0} + \mathbf{X}_{2,0} + \mathbf{X}_{3,1} + \mathbf{X}_{4,1} + \mathbf{X}_{5,0} + \mathbf{X}_{6,0} \to \mathbf{Y}_1$$
$$\mathbf{X}_{1,0} + \mathbf{X}_{2,0} + \mathbf{X}_{3,1} + \mathbf{X}_{4,1} + \mathbf{X}_{5,0} + \mathbf{X}_{6,1} \to \mathbf{Y}_1$$
$$\mathbf{X}_{1,0} + \mathbf{X}_{2,0} + \mathbf{X}_{3,1} + \mathbf{X}_{4,1} + \mathbf{X}_{5,1} + \mathbf{X}_{6,0} \to \mathbf{Y}_1$$
$$\mathbf{X}_{1,0} + \mathbf{X}_{2,0} + \mathbf{X}_{3,1} + \mathbf{X}_{4,1} + \mathbf{X}_{5,1} + \mathbf{X}_{6,1} \to \mathbf{Y}_1$$
$$\mathbf{X}_{1,0} + \mathbf{X}_{2,1} + \mathbf{X}_{3,0} + \mathbf{X}_{4,0} + \mathbf{X}_{5,0} + \mathbf{X}_{6,0} \to \mathbf{Y}_1$$
$$\mathbf{X}_{1,0} + \mathbf{X}_{2,1} + \mathbf{X}_{3,0} + \mathbf{X}_{4,0} + \mathbf{X}_{5,0} + \mathbf{X}_{6,1} \to \mathbf{Y}_1$$
$$\mathbf{X}_{1,0} + \mathbf{X}_{2,1} + \mathbf{X}_{3,0} + \mathbf{X}_{4,0} + \mathbf{X}_{5,1} + \mathbf{X}_{6,0} \to \mathbf{Y}_1$$
$$\mathbf{X}_{1,0} + \mathbf{X}_{2,1} + \mathbf{X}_{3,0} + \mathbf{X}_{4,0} + \mathbf{X}_{5,1} + \mathbf{X}_{6,1} \to \mathbf{Y}_1$$
$$\mathbf{X}_{1,0} + \mathbf{X}_{2,1} + \mathbf{X}_{3,0} + \mathbf{X}_{4,1} + \mathbf{X}_{5,0} + \mathbf{X}_{6,0} \to \mathbf{Y}_1$$
$$\mathbf{X}_{1,0} + \mathbf{X}_{2,1} + \mathbf{X}_{3,0} + \mathbf{X}_{4,1} + \mathbf{X}_{5,0} + \mathbf{X}_{6,1} \to \mathbf{Y}_1$$
$$\mathbf{X}_{1,0} + \mathbf{X}_{2,1} + \mathbf{X}_{3,0} + \mathbf{X}_{4,1} + \mathbf{X}_{5,1} + \mathbf{X}_{6,0} \to \mathbf{Y}_1$$
$$\mathbf{X}_{1,0} + \mathbf{X}_{2,1} + \mathbf{X}_{3,0} + \mathbf{X}_{4,1} + \mathbf{X}_{5,1} + \mathbf{X}_{6,1} \to \mathbf{Y}_1$$

$$\mathbf{X}_{1,0} + \mathbf{X}_{2,1} + \mathbf{X}_{3,1} + \mathbf{X}_{4,0} + \mathbf{X}_{5,0} + \mathbf{X}_{6,0} \rightarrow \mathbf{Y}_1$$
$$\mathbf{X}_{1,0} + \mathbf{X}_{2,1} + \mathbf{X}_{3,1} + \mathbf{X}_{4,0} + \mathbf{X}_{5,0} + \mathbf{X}_{6,1} \rightarrow \mathbf{Y}_1$$
$$\mathbf{X}_{1,0} + \mathbf{X}_{2,1} + \mathbf{X}_{3,1} + \mathbf{X}_{4,0} + \mathbf{X}_{5,1} + \mathbf{X}_{6,0} \rightarrow \mathbf{Y}_1$$
$$\mathbf{X}_{1,0} + \mathbf{X}_{2,1} + \mathbf{X}_{3,1} + \mathbf{X}_{4,0} + \mathbf{X}_{5,1} + \mathbf{X}_{6,1} \rightarrow \mathbf{Y}_1$$
$$\mathbf{X}_{1,0} + \mathbf{X}_{2,1} + \mathbf{X}_{3,1} + \mathbf{X}_{4,1} + \mathbf{X}_{5,0} + \mathbf{X}_{6,0} \rightarrow \mathbf{Y}_1$$
$$\mathbf{X}_{1,0} + \mathbf{X}_{2,1} + \mathbf{X}_{3,1} + \mathbf{X}_{4,1} + \mathbf{X}_{5,0} + \mathbf{X}_{6,1} \rightarrow \mathbf{Y}_1$$
$$\mathbf{X}_{1,0} + \mathbf{X}_{2,1} + \mathbf{X}_{3,1} + \mathbf{X}_{4,1} + \mathbf{X}_{5,1} + \mathbf{X}_{6,0} \rightarrow \mathbf{Y}_1$$
$$\mathbf{X}_{1,0} + \mathbf{X}_{2,1} + \mathbf{X}_{3,1} + \mathbf{X}_{4,1} + \mathbf{X}_{5,1} + \mathbf{X}_{6,1} \rightarrow \mathbf{Y}_1$$
$$\mathbf{X}_{1,1} + \mathbf{X}_{2,0} + \mathbf{X}_{3,0} + \mathbf{X}_{4,0} + \mathbf{X}_{5,0} + \mathbf{X}_{6,0} \rightarrow \mathbf{Y}_1$$
$$\mathbf{X}_{1,1} + \mathbf{X}_{2,0} + \mathbf{X}_{3,0} + \mathbf{X}_{4,0} + \mathbf{X}_{5,0} + \mathbf{X}_{6,1} \rightarrow \mathbf{Y}_1$$
$$\mathbf{X}_{1,1} + \mathbf{X}_{2,0} + \mathbf{X}_{3,0} + \mathbf{X}_{4,0} + \mathbf{X}_{5,1} + \mathbf{X}_{6,0} \rightarrow \mathbf{Y}_1$$
$$\mathbf{X}_{1,1} + \mathbf{X}_{2,0} + \mathbf{X}_{3,0} + \mathbf{X}_{4,0} + \mathbf{X}_{5,1} + \mathbf{X}_{6,1} \rightarrow \mathbf{Y}_1$$
$$\mathbf{X}_{1,1} + \mathbf{X}_{2,0} + \mathbf{X}_{3,0} + \mathbf{X}_{4,1} + \mathbf{X}_{5,0} + \mathbf{X}_{6,0} \rightarrow \mathbf{Y}_1$$
$$\mathbf{X}_{1,1} + \mathbf{X}_{2,0} + \mathbf{X}_{3,0} + \mathbf{X}_{4,1} + \mathbf{X}_{5,0} + \mathbf{X}_{6,1} \rightarrow \mathbf{Y}_1$$
$$\mathbf{X}_{1,1} + \mathbf{X}_{2,0} + \mathbf{X}_{3,0} + \mathbf{X}_{4,1} + \mathbf{X}_{5,1} + \mathbf{X}_{6,0} \rightarrow \mathbf{Y}_1$$
$$\mathbf{X}_{1,1} + \mathbf{X}_{2,0} + \mathbf{X}_{3,0} + \mathbf{X}_{4,1} + \mathbf{X}_{5,1} + \mathbf{X}_{6,1} \rightarrow \mathbf{Y}_1$$
$$\mathbf{X}_{1,1} + \mathbf{X}_{2,0} + \mathbf{X}_{3,1} + \mathbf{X}_{4,0} + \mathbf{X}_{5,0} + \mathbf{X}_{6,0} \rightarrow \mathbf{Y}_1$$
$$\mathbf{X}_{1,1} + \mathbf{X}_{2,0} + \mathbf{X}_{3,1} + \mathbf{X}_{4,0} + \mathbf{X}_{5,0} + \mathbf{X}_{6,1} \rightarrow \mathbf{Y}_1$$
$$\mathbf{X}_{1,1} + \mathbf{X}_{2,0} + \mathbf{X}_{3,1} + \mathbf{X}_{4,0} + \mathbf{X}_{5,1} + \mathbf{X}_{6,0} \rightarrow \mathbf{Y}_1$$
$$\mathbf{X}_{1,1} + \mathbf{X}_{2,0} + \mathbf{X}_{3,1} + \mathbf{X}_{4,0} + \mathbf{X}_{5,1} + \mathbf{X}_{6,1} \rightarrow \mathbf{Y}_1$$
$$\mathbf{X}_{1,1} + \mathbf{X}_{2,0} + \mathbf{X}_{3,1} + \mathbf{X}_{4,1} + \mathbf{X}_{5,0} + \mathbf{X}_{6,0} \rightarrow \mathbf{Y}_1$$
$$\mathbf{X}_{1,1} + \mathbf{X}_{2,0} + \mathbf{X}_{3,1} + \mathbf{X}_{4,1} + \mathbf{X}_{5,0} + \mathbf{X}_{6,1} \rightarrow \mathbf{Y}_1$$
$$\mathbf{X}_{1,1} + \mathbf{X}_{2,0} + \mathbf{X}_{3,1} + \mathbf{X}_{4,1} + \mathbf{X}_{5,1} + \mathbf{X}_{6,0} \rightarrow \mathbf{Y}_1$$
$$\mathbf{X}_{1,1} + \mathbf{X}_{2,0} + \mathbf{X}_{3,1} + \mathbf{X}_{4,1} + \mathbf{X}_{5,1} + \mathbf{X}_{6,1} \rightarrow \mathbf{Y}_1$$
$$\mathbf{X}_{1,1} + \mathbf{X}_{2,1} + \mathbf{X}_{3,0} + \mathbf{X}_{4,0} + \mathbf{X}_{5,0} + \mathbf{X}_{6,0} \rightarrow \mathbf{Y}_1$$
$$\mathbf{X}_{1,1} + \mathbf{X}_{2,1} + \mathbf{X}_{3,0} + \mathbf{X}_{4,0} + \mathbf{X}_{5,0} + \mathbf{X}_{6,1} \rightarrow \mathbf{Y}_1$$
$$\mathbf{X}_{1,1} + \mathbf{X}_{2,1} + \mathbf{X}_{3,0} + \mathbf{X}_{4,0} + \mathbf{X}_{5,1} + \mathbf{X}_{6,0} \rightarrow \mathbf{Y}_1$$
$$\mathbf{X}_{1,1} + \mathbf{X}_{2,1} + \mathbf{X}_{3,0} + \mathbf{X}_{4,0} + \mathbf{X}_{5,1} + \mathbf{X}_{6,1} \rightarrow \mathbf{Y}_1$$
$$\mathbf{X}_{1,1} + \mathbf{X}_{2,1} + \mathbf{X}_{3,0} + \mathbf{X}_{4,1} + \mathbf{X}_{5,0} + \mathbf{X}_{6,0} \rightarrow \mathbf{Y}_1$$
$$\mathbf{X}_{1,1} + \mathbf{X}_{2,1} + \mathbf{X}_{3,0} + \mathbf{X}_{4,1} + \mathbf{X}_{5,0} + \mathbf{X}_{6,1} \rightarrow \mathbf{Y}_1$$
$$\mathbf{X}_{1,1} + \mathbf{X}_{2,1} + \mathbf{X}_{3,0} + \mathbf{X}_{4,1} + \mathbf{X}_{5,1} + \mathbf{X}_{6,0} \rightarrow \mathbf{Y}_1$$
$$\mathbf{X}_{1,1} + \mathbf{X}_{2,1} + \mathbf{X}_{3,0} + \mathbf{X}_{4,1} + \mathbf{X}_{5,1} + \mathbf{X}_{6,1} \rightarrow \mathbf{Y}_1$$
$$\mathbf{X}_{1,1} + \mathbf{X}_{2,1} + \mathbf{X}_{3,1} + \mathbf{X}_{4,0} + \mathbf{X}_{5,0} + \mathbf{X}_{6,0} \rightarrow \mathbf{Y}_0$$
$$\mathbf{X}_{1,1} + \mathbf{X}_{2,1} + \mathbf{X}_{3,1} + \mathbf{X}_{4,0} + \mathbf{X}_{5,0} + \mathbf{X}_{6,1} \rightarrow \mathbf{Y}_0$$
$$\mathbf{X}_{1,1} + \mathbf{X}_{2,1} + \mathbf{X}_{3,1} + \mathbf{X}_{4,0} + \mathbf{X}_{5,1} + \mathbf{X}_{6,0} \rightarrow \mathbf{Y}_0$$
$$\mathbf{X}_{1,1} + \mathbf{X}_{2,1} + \mathbf{X}_{3,1} + \mathbf{X}_{4,0} + \mathbf{X}_{5,1} + \mathbf{X}_{6,1} \rightarrow \mathbf{Y}_0$$
$$\mathbf{X}_{1,1} + \mathbf{X}_{2,1} + \mathbf{X}_{3,1} + \mathbf{X}_{4,1} + \mathbf{X}_{5,0} + \mathbf{X}_{6,0} \rightarrow \mathbf{Y}_0$$
$$\mathbf{X}_{1,1} + \mathbf{X}_{2,1} + \mathbf{X}_{3,1} + \mathbf{X}_{4,1} + \mathbf{X}_{5,0} + \mathbf{X}_{6,1} \rightarrow \mathbf{Y}_0$$
$$\mathbf{X}_{1,1} + \mathbf{X}_{2,1} + \mathbf{X}_{3,1} + \mathbf{X}_{4,1} + \mathbf{X}_{5,1} + \mathbf{X}_{6,0} \rightarrow \mathbf{Y}_0$$
$$\mathbf{X}_{1,1} + \mathbf{X}_{2,1} + \mathbf{X}_{3,1} + \mathbf{X}_{4,1} + \mathbf{X}_{5,1} + \mathbf{X}_{6,1} \rightarrow \mathbf{Y}_1$$
