# Supplementary material for: Computing mathematical functions with chemical reactions via stochastic logic
Source: PLoS One. 2023 May 8;18(5):e0281574. doi: 10.1371/journal.pone.0281574 (PMC10166555; doi:10.1371/journal.pone.0281574)
Supplement: S2 File — We use the NDSolveValue command in Mathematica to simulate the system of differential equations for the 3-input XOR in Section 5. The script includes the various error analyses and image printing commands. (PDF) [file pone.0281574.s002.pdf]

```
(* Define 3 input XOR *)
f[x_, y_, z_] := (1 - x) * (1 - y) * z + (1 - x) * y * (1 - z) + x * (1 - y) * (1 - z) + x * y * z;
f[x_, y_, z_] := (1 - x) * y * (1 - z) + x * (1 - y) * (1 - z) + x * y * z;

(* Define range of stochastic values you want to test for *)
range = {0, 0.1, 0.2, 0.3, 0.4, 0.5, 0.6, 0.7, 0.8, 0.9, 1};
m = Length[range];
(* Create 3D matrices to hold values *)
temp = ConstantArray[0, m];
temp1 = ConstantArray[temp, m];
(* fpre (predicted) assumes no error *)
fpre = ConstantArray[temp1, m];
(* fexp (experimental) solves via diff eqs given a rate *)
fexp = ConstantArray[temp1, m];
(* fmatrix stores the difference for each cell i.e. the error *)
fmatrix = ConstantArray[temp1, m];

(* calculate proper matrix for F *)
For[i = 1, i < m + 1, i++,
 For[j = 1, j < m + 1, j++,
  For[k = 1, k < m + 1, k++,
   x = range[[i]];
   y = range[[j]];
   z = range[[k]];
   fpre[[i, j, k]] = f[x, y, z]
  ]
 ]
]

(* start with same rates *)
rates = ConstantArray[10, 8];
(* k1 is scaled by 10 *)
k1 = 10 * rates[[1]]; k2 = rates[[2]]; k3 = rates[[3]]; k4 = rates[[4]];
k5 = rates[[5]]; k6 = rates[[6]]; k7 = rates[[7]]; k8 = rates[[8]];

(* Define all diff eqs *)
eqns = {
   x0'[t] == -1 * x0[t] *
     (k1 * y0[t] * z0[t] + k2 * y0[t] * z1[t] + k3 * y1[t] * z0[t] + k4 * y1[t] * z1[t]),
   x1'[t] == -1 * x1[t] *
     (k5 * y0[t] * z0[t] + k6 * y0[t] * z1[t] + k7 * y1[t] * z0[t] + k8 * y1[t] * z1[t]),
   y0'[t] == -1 * y0[t] *
     (k1 * x0[t] * z0[t] + k2 * x0[t] * z1[t] + k5 * x1[t] * z0[t] + k6 * x1[t] * z1[t]),
   y1'[t] == -1 * y1[t] *
     (k3 * x0[t] * z0[t] + k4 * x0[t] * z1[t] + k7 * x1[t] * z0[t] + k8 * x1[t] * z1[t]),
   z0'[t] == -1 * z0[t] *
```

```
      (k1 * x0[t] * y0[t] + k3 * x0[t] * y1[t] + k5 * x1[t] * y0[t] + k7 * x1[t] * y1[t]),
    z1'[t] == -1 * z1[t] *
      (k2 * x0[t] * y0[t] + k4 * x0[t] * y1[t] + k6 * x1[t] * y0[t] + k8 * x1[t] * y1[t]),
    f0'[t] ==
     k1 * x0[t] * y0[t] * z0[t] + k2 * x0[t] * y0[t] * z1[t] +
      k4 * x0[t] * y1[t] * z1[t] + k6 * x1[t] * y0[t] * z1[t] + k7 * x1[t] * y1[t] * z0[t],
    f0[0] == 0,
    f1'[t] ==
     k3 * x0[t] * y1[t] * z0[t] + k5 * x1[t] * y0[t] * z0[t] + k8 * x1[t] * y1[t] * z1[t],
    f1[0] == 0
   };
(* Define output functions *)
funcs = {f0, f1};
(* Define time range to integrate over *)
trange = {t, 0, 100};

(* Solve the fexp matrix *)
For[i = 1, i < m + 1, i++,
 For[j = 1, j < m + 1, j++,
  For[k = 1, k < m + 1, k++,
    x = range[[i]];
    y = range[[j]];
    z = range[[k]];
    (* for each case find the new initial conditions *)
    conds = {
      x0[0] == 100 * (1 - x),
      x1[0] == 100 * (x),
      y0[0] == 100 * (1 - y),
      y1[0] == 100 * (y),
      z0[0] == 100 * (1 - z),
      z1[0] == 100 * (z)
     };
    s = NDSolveValue[Join[eqns, conds], funcs, trange];
    (* find f0 and f1 and compute f *)
    a = s[[1]][100]; b = s[[2]][100];
    fexp[[i, j, k]] = b / (b + a);
   ]
  ]
]

(* compute the difference error for each cell *)
For[i = 1, i < m + 1, i++,
 For[j = 1, j < m + 1, j++,
  For[k = 1, k < m + 1, k++,
    fmatrix[[i, j, k]] = Abs[fexp[[i, j, k]] - fpre[[i, j, k]]]
   ]
  ]
```

```
]

(* flatten to find the largest error *)
check = Flatten[fmatrix];
max = Max[check]
(* list of form {x,y,z,error(x,y,z)} to plot *)
data = {};
For[i = 1, i < m + 1, i++,
 For[j = 1, j < m + 1, j++,
  For[k = 1, k < m + 1, k++,
   x = range〚i〛;
   y = range〚j〛;
   z = range〚k〛;
   data = Insert[data, {x, y, z, fmatrix〚i, j, k〛 / max}, -1];
  ]
 ]
]
mycolor = ColorData[{"DeepSeaColors", "Reverse"}];
Graphics3D[{Opacity[0.8], PointSize[0.03],
  Point[data〚All, 1 ;; 3〛 , VertexColors → mycolor /@ data〚All, 4〛 ]},
 Axes → True, AxesLabel → {"x", "y", "z"},
 LabelStyle → Directive[Bold, Medium], BoxRatios → {1, 1, 1}, ImageSize → Large]
BarLegend[{"DeepSeaColors", "Reverse"}, "Ticks" → {{0, 0}, {1, max}}]
```

```
0.310836
```

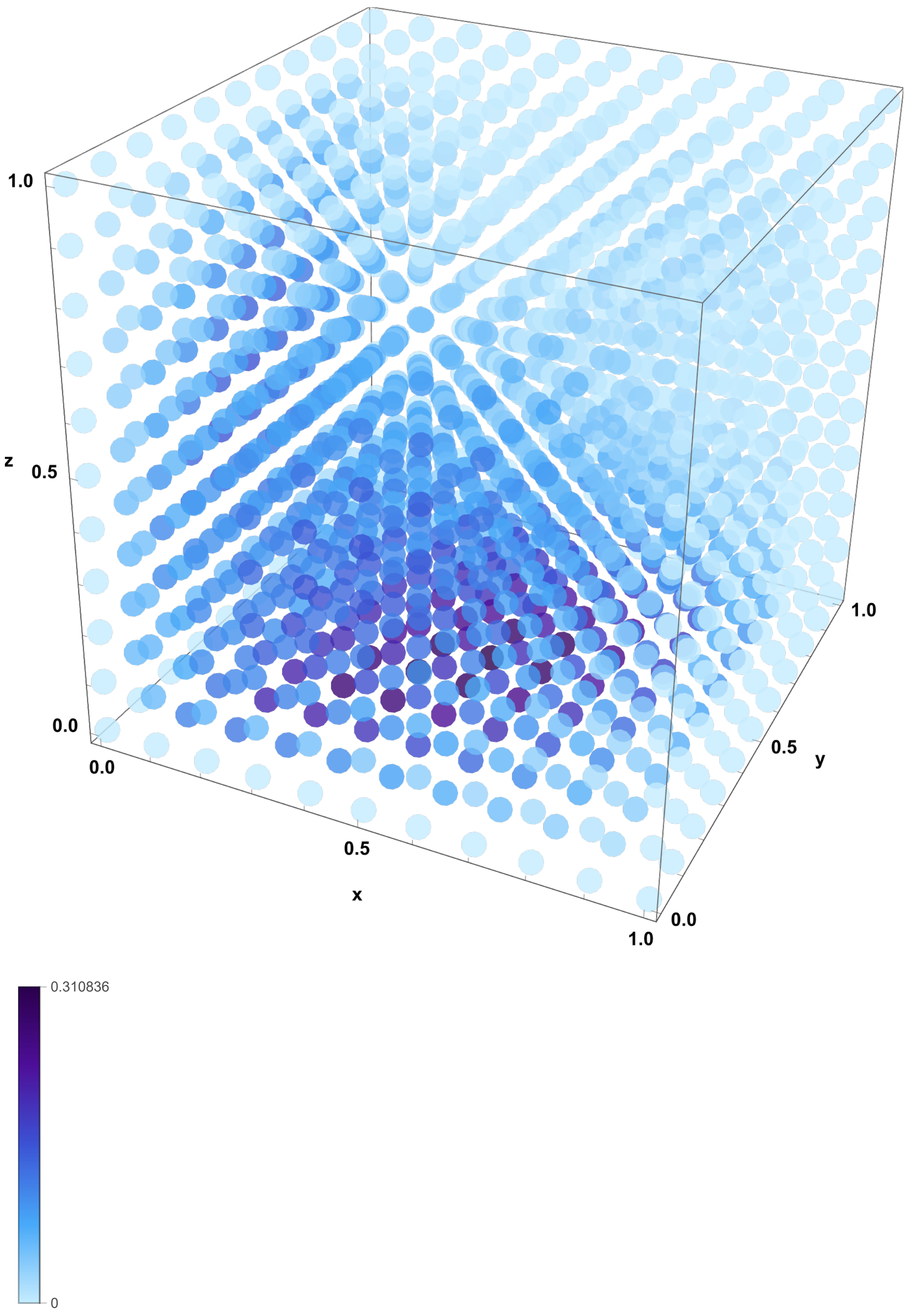
1.0
z
0.5
0.0
0.0
0.5
x
1.0
0.0
0.5
y
1.0
0.310836
0

```
(* flatten to find the largest error *)
check = Flatten[fmatrix];
max = Max[check]
(* list of form {x,y,z,error(x,y,z)} to plot *)
data = {};
For[i = 1, i < m + 1, i++,
 For[j = 1, j < m + 1, j++,
  For[k = 1, k < m + 1, k++,
   x = range[[i]];
   y = range[[j]];
   z = range[[k]];
   data = Insert[data, {x, y, z, fmatrix[[i, j, k]] / max}, -1];
  ]
 ]
]
mycolor = ColorData[{"DeepSeaColors", "Reverse"}];
Graphics3D[{Opacity[0.8], PointSize[0.03],
  Point[data[[All, 1 ;; 3]] , VertexColors → mycolor /@ data[[All, 4]] ]},
 Axes → True, BoxRatios → {1, 1, 1}]
BarLegend[{"DeepSeaColors", "Reverse"}, LegendLabel → None]
```

```
0.127169
```

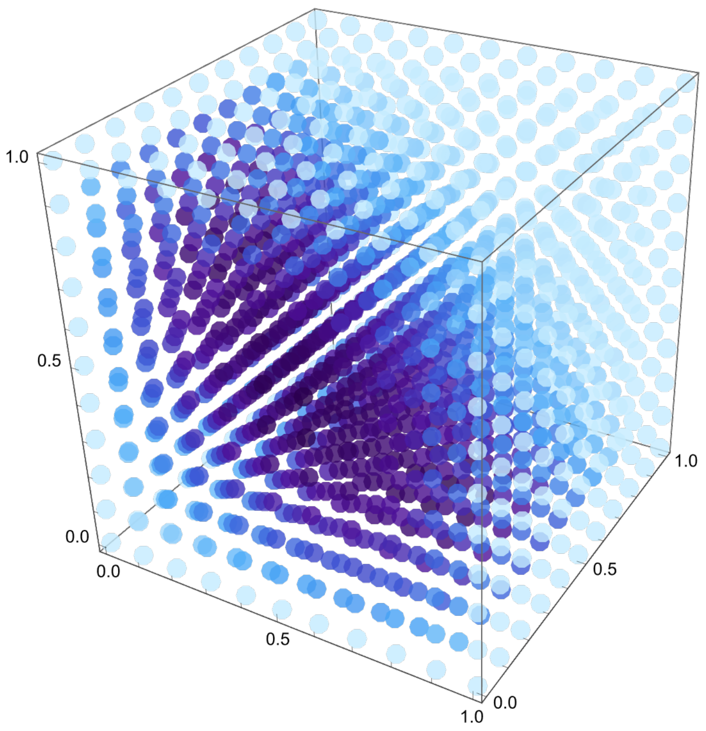

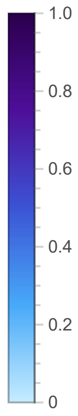


```
(* start with same rates *)
rates = ConstantArray[10, 8];
(* k1 is scaled down by 10 *)
k1 = rates[[1]] / 10; k2 = rates[[2]]; k3 = rates[[3]]; k4 = rates[[4]];
k5 = rates[[5]]; k6 = rates[[6]]; k7 = rates[[7]]; k8 = rates[[8]];

(* Define all diff eqs *)
eqns = {
   x0'[t] == -1 * x0[t] *
     (k1 * y0[t] * z0[t] + k2 * y0[t] * z1[t] + k3 * y1[t] * z0[t] + k4 * y1[t] * z1[t]),
```

```
   x1'[t] == -1 * x1[t] *
     (k5 * y0[t] * z0[t] + k6 * y0[t] * z1[t] + k7 * y1[t] * z0[t] + k8 * y1[t] * z1[t]),
   y0'[t] == -1 * y0[t] *
     (k1 * x0[t] * z0[t] + k2 * x0[t] * z1[t] + k5 * x1[t] * z0[t] + k6 * x1[t] * z1[t]),
   y1'[t] == -1 * y1[t] *
     (k3 * x0[t] * z0[t] + k4 * x0[t] * z1[t] + k7 * x1[t] * z0[t] + k8 * x1[t] * z1[t]),
   z0'[t] ==  -1 * z0[t] *
     (k1 * x0[t] * y0[t] + k3 * x0[t] * y1[t] + k5 * x1[t] * y0[t] + k7 * x1[t] * y1[t]),
   z1'[t] == -1 * z1[t] *
     (k2 * x0[t] * y0[t] + k4 * x0[t] * y1[t] + k6 * x1[t] * y0[t] + k8 * x1[t] * y1[t]),
   f0'[t] ==
    k1 * x0[t] * y0[t] * z0[t] + k4 * x0[t] * y1[t] * z1[t] +
     k6 * x1[t] * y0[t] * z1[t] + k7 * x1[t] * y1[t] * z0[t],
   f0[0] == 0,
   f1'[t] ==
    k2 * x0[t] * y0[t] * z1[t] + k3 * x0[t] * y1[t] * z0[t] +
     k5 * x1[t] * y0[t] * z0[t] + k8 * x1[t] * y1[t] * z1[t],
   f1[0] == 0
  };
(* Define output functions *)
funcs = {f0, f1};
(* Define time range to integrate over *)
trange = {t, 0, 100};

(* Solve the fexp matrix *)
For[i = 1, i < m + 1, i++,
 For[j = 1, j < m + 1, j++,
  For[k = 1, k < m + 1, k++,
   x = range[[i]];
   y = range[[j]];
   z = range[[k]];
   (* for each case find the new initial conditions *)
   conds = {
     x0[0] == 100 * (1 - x),
     x1[0] == 100 * (x),
     y0[0] == 100 * (1 - y),
     y1[0] == 100 * (y),
     z0[0] == 100 * (1 - z),
     z1[0] == 100 * (z)
    };
   s = NDSolveValue[Join[eqns, conds], funcs, trange];
   (* find f0 and f1 and compute f *)
   a = s[[1]][100]; b = s[[2]][100];
   fexp[[i, j, k]] = b / (b + a);
  ]
 ]
]
```

```
(* compute the difference error for each cell *)
For[i = 1, i < m + 1, i++,
 For[j = 1, j < m + 1, j++,
  For[k = 1, k < m + 1, k++,
   fmatrix⟦i, j, k⟧ = Abs[fexp⟦i, j, k⟧ - fpre⟦i, j, k⟧]
  ]
 ]
]
(* flatten to find the largest error *)
check = Flatten[fmatrix];
max = Max[check]
(* list of form {x,y,z,error(x,y,z)} to plot *)
data = {};
For[i = 1, i < m + 1, i++,
 For[j = 1, j < m + 1, j++,
  For[k = 1, k < m + 1, k++,
   x = range⟦i⟧;
   y = range⟦j⟧;
   z = range⟦k⟧;
   data = Insert[data, {x, y, z, fmatrix⟦i, j, k⟧ / max}, -1];
  ]
 ]
]
mycolor = ColorData["CMYKColors"];
Graphics3D[{Opacity[0.8], PointSize[0.03],
  Point[data⟦All, 1 ;; 3⟧ , VertexColors → mycolor /@ data⟦All, 4⟧ ]},
 Axes → True, BoxRatios → {1, 1, 1}]
BarLegend["CMYKColors", LegendLabel → None]
```

0.127169

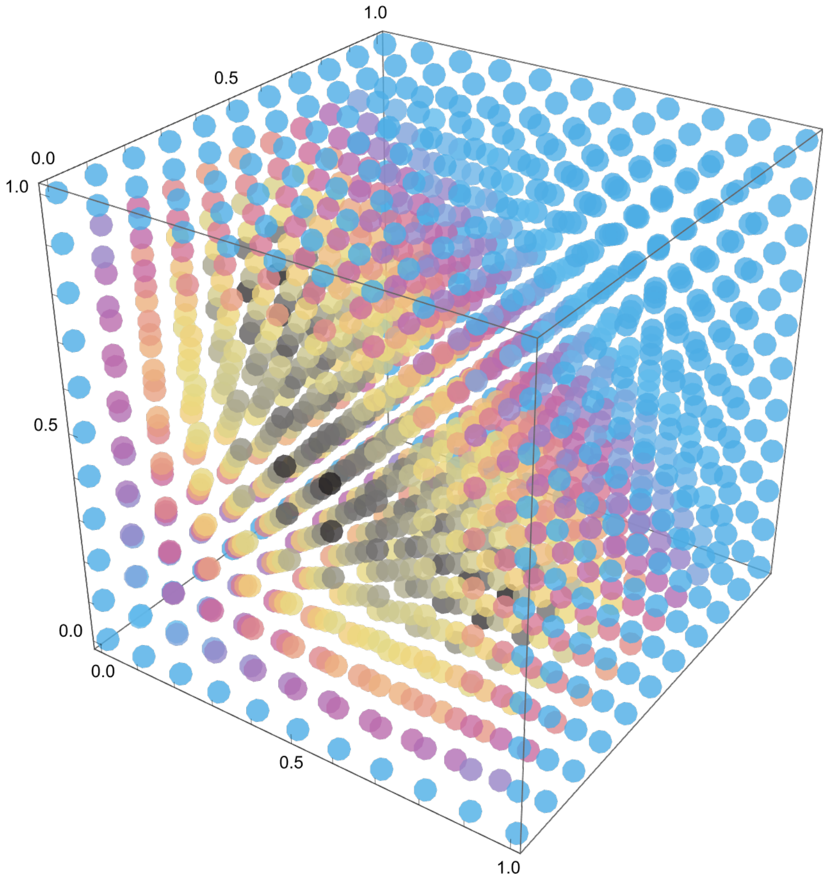


```
(* start with same rates *)
rates = ConstantArray[10, 8];
(* k8 is scaled by 10 *)
k1 = rates[[1]]; k2 = rates[[2]]; k3 = rates[[3]]; k4 = rates[[4]];
k5 = rates[[5]]; k6 = rates[[6]]; k7 = rates[[7]]; k8 = rates[[8]] * 10;

(* Define all diff eqs *)
eqns = {
   x0'[t] == -1 * x0[t] *
     (k1 * y0[t] * z0[t] + k2 * y0[t] * z1[t] + k3 * y1[t] * z0[t] + k4 * y1[t] * z1[t]),
   x1'[t] == -1 * x1[t] *
     (k5 * y0[t] * z0[t] + k6 * y0[t] * z1[t] + k7 * y1[t] * z0[t] + k8 * y1[t] * z1[t]),
   y0'[t] == -1 * y0[t] *
     (k1 * x0[t] * z0[t] + k2 * x0[t] * z1[t] + k5 * x1[t] * z0[t] + k6 * x1[t] * z1[t]),
   y1'[t] == -1 * y1[t] *
     (k3 * x0[t] * z0[t] + k4 * x0[t] * z1[t] + k7 * x1[t] * z0[t] + k8 * x1[t] * z1[t]),
   z0'[t] ==  -1 * z0[t] *
     (k1 * x0[t] * y0[t] + k3 * x0[t] * y1[t] + k5 * x1[t] * y0[t] + k7 * x1[t] * y1[t]),
   z1'[t] == -1 * z1[t] *
     (k2 * x0[t] * y0[t] + k4 * x0[t] * y1[t] + k6 * x1[t] * y0[t] + k8 * x1[t] * y1[t]),
```

```
   f0'[t] ==
    k1 * x0[t] * y0[t] * z0[t] + k4 * x0[t] * y1[t] * z1[t] +
     k6 * x1[t] * y0[t] * z1[t] + k7 * x1[t] * y1[t] * z0[t],
   f0[0] == 0,
   f1'[t] ==
    k2 * x0[t] * y0[t] * z1[t] + k3 * x0[t] * y1[t] * z0[t] +
     k5 * x1[t] * y0[t] * z0[t] + k8 * x1[t] * y1[t] * z1[t],
   f1[0] == 0
  };
(* Define output functions *)
funcs = {f0, f1};
(* Define time range to integrate over *)
trange = {t, 0, 100};

(* Solve the fexp matrix *)
For[i = 1, i < m + 1, i++,
 For[j = 1, j < m + 1, j++,
  For[k = 1, k < m + 1, k++,
   x = range[[i]];
   y = range[[j]];
   z = range[[k]];
   (* for each case find the new initial conditions *)
   conds = {
     x0[0] == 100 * (1 - x),
     x1[0] == 100 * (x),
     y0[0] == 100 * (1 - y),
     y1[0] == 100 * (y),
     z0[0] == 100 * (1 - z),
     z1[0] == 100 * (z)
    };
   s = NDSolveValue[Join[eqns, conds], funcs, trange];
   (* find f0 and f1 and compute f *)
   a = s[[1]][100]; b = s[[2]][100];
   fexp[[i, j, k]] = b / (b + a);
  ]
 ]
]
(* compute the difference error for each cell *)
For[i = 1, i < m + 1, i++,
 For[j = 1, j < m + 1, j++,
  For[k = 1, k < m + 1, k++,
   fmatrix[[i, j, k]] = Abs[fexp[[i, j, k]] - fpre[[i, j, k]]]
  ]
 ]
]
(* flatten to find the largest error *)
check = Flatten[fmatrix];
```

```
max = Max[check]
(* list of form {x,y,z,error(x,y,z)} to plot *)
data = {};
For[i = 1, i < m + 1, i++,
 For[j = 1, j < m + 1, j++,
  For[k = 1, k < m + 1, k++,
   x = range[[i]];
   y = range[[j]];
   z = range[[k]];
   data = Insert[data, {x, y, z, fmatrix[[i, j, k]] / max}, -1];
  ]
 ]
]
mycolor = ColorData["CMYKColors"];
Graphics3D[
 {PointSize[Large], Point[data[[All, 1 ;; 3]] , VertexColors → mycolor /@ data[[All, 4]] ]},
 Axes → True, BoxRatios → {1, 1, 1}]
BarLegend["CMYKColors", LegendLabel → None]
```

0.310836

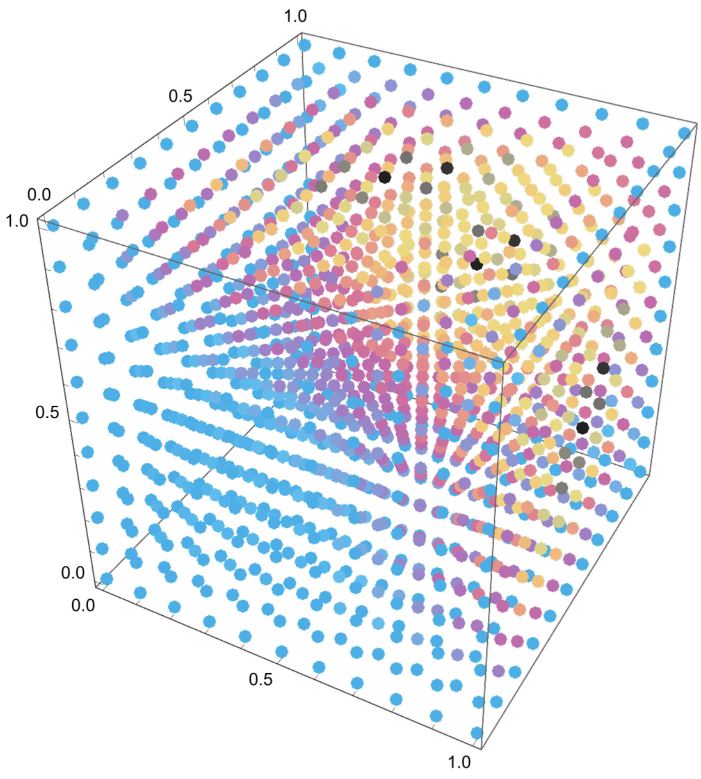

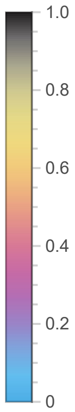


```
(* start with same rates *)
rates = ConstantArray[10, 8];
(* k8 is scaled down by 10 *)
k1 = rates⟦1⟧; k2 = rates⟦2⟧; k3 = rates⟦3⟧; k4 = rates⟦4⟧;
k5 = rates⟦5⟧; k6 = rates⟦6⟧; k7 = rates⟦7⟧; k8 = rates⟦8⟧ / 10;

(* Define all diff eqs *)
eqns = {
   x0'[t] == -1 * x0[t] *
     (k1 * y0[t] * z0[t] + k2 * y0[t] * z1[t] + k3 * y1[t] * z0[t] + k4 * y1[t] * z1[t]),
   x1'[t] == -1 * x1[t] *
     (k5 * y0[t] * z0[t] + k6 * y0[t] * z1[t] + k7 * y1[t] * z0[t] + k8 * y1[t] * z1[t]),
   y0'[t] == -1 * y0[t] *
     (k1 * x0[t] * z0[t] + k2 * x0[t] * z1[t] + k5 * x1[t] * z0[t] + k6 * x1[t] * z1[t]),
   y1'[t] == -1 * y1[t] *
     (k3 * x0[t] * z0[t] + k4 * x0[t] * z1[t] + k7 * x1[t] * z0[t] + k8 * x1[t] * z1[t]),
   z0'[t] ==  -1 * z0[t] *
     (k1 * x0[t] * y0[t] + k3 * x0[t] * y1[t] + k5 * x1[t] * y0[t] + k7 * x1[t] * y1[t]),
   z1'[t] == -1 * z1[t] *
     (k2 * x0[t] * y0[t] + k4 * x0[t] * y1[t] + k6 * x1[t] * y0[t] + k8 * x1[t] * y1[t]),
   f0'[t] ==
    k1 * x0[t] * y0[t] * z0[t] + k4 * x0[t] * y1[t] * z1[t] +
     k6 * x1[t] * y0[t] * z1[t] + k7 * x1[t] * y1[t] * z0[t],
   f0[0] == 0,
   f1'[t] ==
    k2 * x0[t] * y0[t] * z1[t] + k3 * x0[t] * y1[t] * z0[t] +
     k5 * x1[t] * y0[t] * z0[t] + k8 * x1[t] * y1[t] * z1[t],
   f1[0] == 0
  };
(* Define output functions *)
funcs = {f0, f1};
(* Define time range to integrate over *)
```

```
trange = {t, 0, 100};

(* Solve the fexp matrix *)
For[i = 1, i < m + 1, i++,
 For[j = 1, j < m + 1, j++,
  For[k = 1, k < m + 1, k++,
   x = range[[i]];
   y = range[[j]];
   z = range[[k]];
   (* for each case find the new initial conditions *)
   conds = {
     x0[0] == 100 * (1 - x),
     x1[0] == 100 * (x),
     y0[0] == 100 * (1 - y),
     y1[0] == 100 * (y),
     z0[0] == 100 * (1 - z),
     z1[0] == 100 * (z)
    };
   s = NDSolveValue[Join[eqns, conds], funcs, trange];
   (* find f0 and f1 and compute f *)
   a = s[[1]][100]; b = s[[2]][100];
   fexp[[i, j, k]] = b / (b + a);
  ]
 ]
]
(* compute the difference error for each cell *)
For[i = 1, i < m + 1, i++,
 For[j = 1, j < m + 1, j++,
  For[k = 1, k < m + 1, k++,
   fmatrix[[i, j, k]] = Abs[fexp[[i, j, k]] - fpre[[i, j, k]]]
  ]
 ]
]
(* flatten to find the largest error *)
check = Flatten[fmatrix];
max = Max[check]
(* list of form {x,y,z,error(x,y,z)} to plot *)
data = {};
For[i = 1, i < m + 1, i++,
 For[j = 1, j < m + 1, j++,
  For[k = 1, k < m + 1, k++,
   x = range[[i]];
   y = range[[j]];
   z = range[[k]];
   data = Insert[data, {x, y, z, fmatrix[[i, j, k]] / max}, -1];
  ]
 ]
```

```
]
mycolor = ColorData["CMYKColors"];
Graphics3D[
 {PointSize[Large], Point[data⟦All, 1 ;; 3⟧  , VertexColors → mycolor /@ data⟦All, 4⟧ ]},
 Axes → True, BoxRatios → {1, 1, 1}]
BarLegend["CMYKColors", LegendLabel → None]
```

0.127169

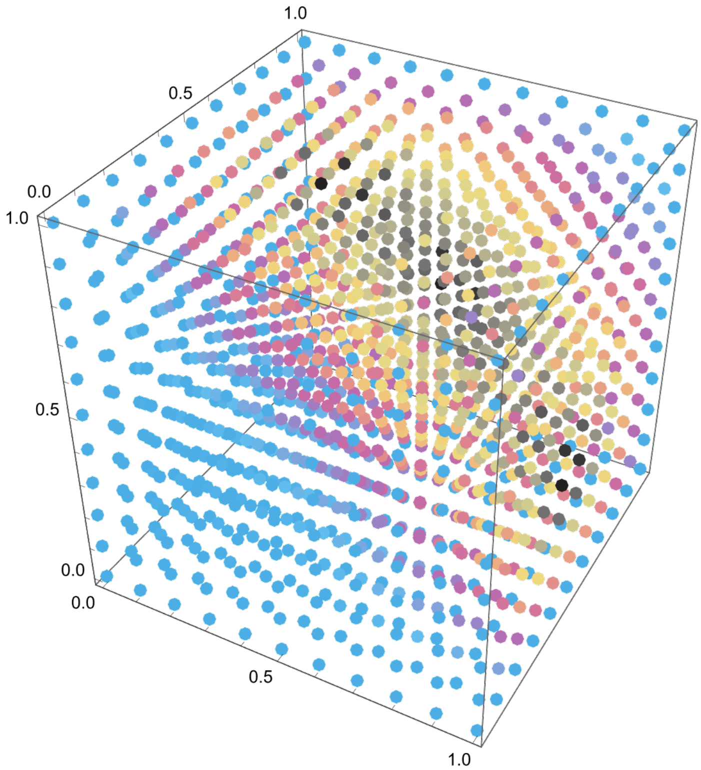

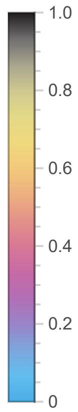


```
(* start with same rates *)
rates = ConstantArray[10, 8];
(* k1 is scaled by 100 *)
```

```
k1 = rates⟦1⟧ * 100; k2 = rates⟦2⟧; k3 = rates⟦3⟧; k4 = rates⟦4⟧;
k5 = rates⟦5⟧; k6 = rates⟦6⟧; k7 = rates⟦7⟧; k8 = rates⟦8⟧;

(* Define all diff eqs *)
eqns = {
   x0'[t] == -1 * x0[t] *
     (k1 * y0[t] * z0[t] + k2 * y0[t] * z1[t] + k3 * y1[t] * z0[t] + k4 * y1[t] * z1[t]),
   x1'[t] == -1 * x1[t] *
     (k5 * y0[t] * z0[t] + k6 * y0[t] * z1[t] + k7 * y1[t] * z0[t] + k8 * y1[t] * z1[t]),
   y0'[t] == -1 * y0[t] *
     (k1 * x0[t] * z0[t] + k2 * x0[t] * z1[t] + k5 * x1[t] * z0[t] + k6 * x1[t] * z1[t]),
   y1'[t] == -1 * y1[t] *
     (k3 * x0[t] * z0[t] + k4 * x0[t] * z1[t] + k7 * x1[t] * z0[t] + k8 * x1[t] * z1[t]),
   z0'[t] ==  -1 * z0[t] *
     (k1 * x0[t] * y0[t] + k3 * x0[t] * y1[t] + k5 * x1[t] * y0[t] + k7 * x1[t] * y1[t]),
   z1'[t] == -1 * z1[t] *
     (k2 * x0[t] * y0[t] + k4 * x0[t] * y1[t] + k6 * x1[t] * y0[t] + k8 * x1[t] * y1[t]),
   f0'[t] ==
    k1 * x0[t] * y0[t] * z0[t] + k4 * x0[t] * y1[t] * z1[t] +
     k6 * x1[t] * y0[t] * z1[t] + k7 * x1[t] * y1[t] * z0[t],
   f0[0] == 0,
   f1'[t] ==
    k2 * x0[t] * y0[t] * z1[t] + k3 * x0[t] * y1[t] * z0[t] +
     k5 * x1[t] * y0[t] * z0[t] + k8 * x1[t] * y1[t] * z1[t],
   f1[0] == 0
  };
(* Define output functions *)
funcs = {f0, f1};
(* Define time range to integrate over *)
trange = {t, 0, 100};

(* Solve the fexp matrix *)
For[i = 1, i < m + 1, i++,
 For[j = 1, j < m + 1, j++,
  For[k = 1, k < m + 1, k++,
   x = range⟦i⟧;
   y = range⟦j⟧;
   z = range⟦k⟧;
   (* for each case find the new initial conditions *)
   conds = {
     x0[0] == 100 * (1 - x),
     x1[0] == 100 * (x),
     y0[0] == 100 * (1 - y),
     y1[0] == 100 * (y),
     z0[0] == 100 * (1 - z),
     z1[0] == 100 * (z)
    };
```

```
    s = NDSolveValue[Join[eqns, conds], funcs, trange];
    (* find f0 and f1 and compute f *)
    a = s[[1]][100]; b = s[[2]][100];
    fexp[[i, j, k]] = b / (b + a);
   ]
  ]
 ]
(* compute the difference error for each cell *)
For[i = 1, i < m + 1, i++,
 For[j = 1, j < m + 1, j++,
  For[k = 1, k < m + 1, k++,
   fmatrix[[i, j, k]] = Abs[fexp[[i, j, k]] - fpre[[i, j, k]]]
  ]
 ]
]
(* flatten to find the largest error *)
check = Flatten[fmatrix];
max = Max[check]
(* list of form {x,y,z,error(x,y,z)} to plot *)
data = {};
For[i = 1, i < m + 1, i++,
 For[j = 1, j < m + 1, j++,
  For[k = 1, k < m + 1, k++,
   x = range[[i]];
   y = range[[j]];
   z = range[[k]];
   data = Insert[data, {x, y, z, fmatrix[[i, j, k]] / max}, -1];
  ]
 ]
]
mycolor = ColorData[{"LakeColors", "Reverse"}];
Graphics3D[{Opacity[0.8], PointSize[0.03],
  Point[data[[All, 1 ;; 3]] , VertexColors → mycolor /@ data[[All, 4]] ]},
 Axes → True, BoxRatios → {1, 1, 1}, ImageSize → Large]
BarLegend[{"LakeColors", "Reverse"}, LegendLabel → None]
```

```
0.459793
```

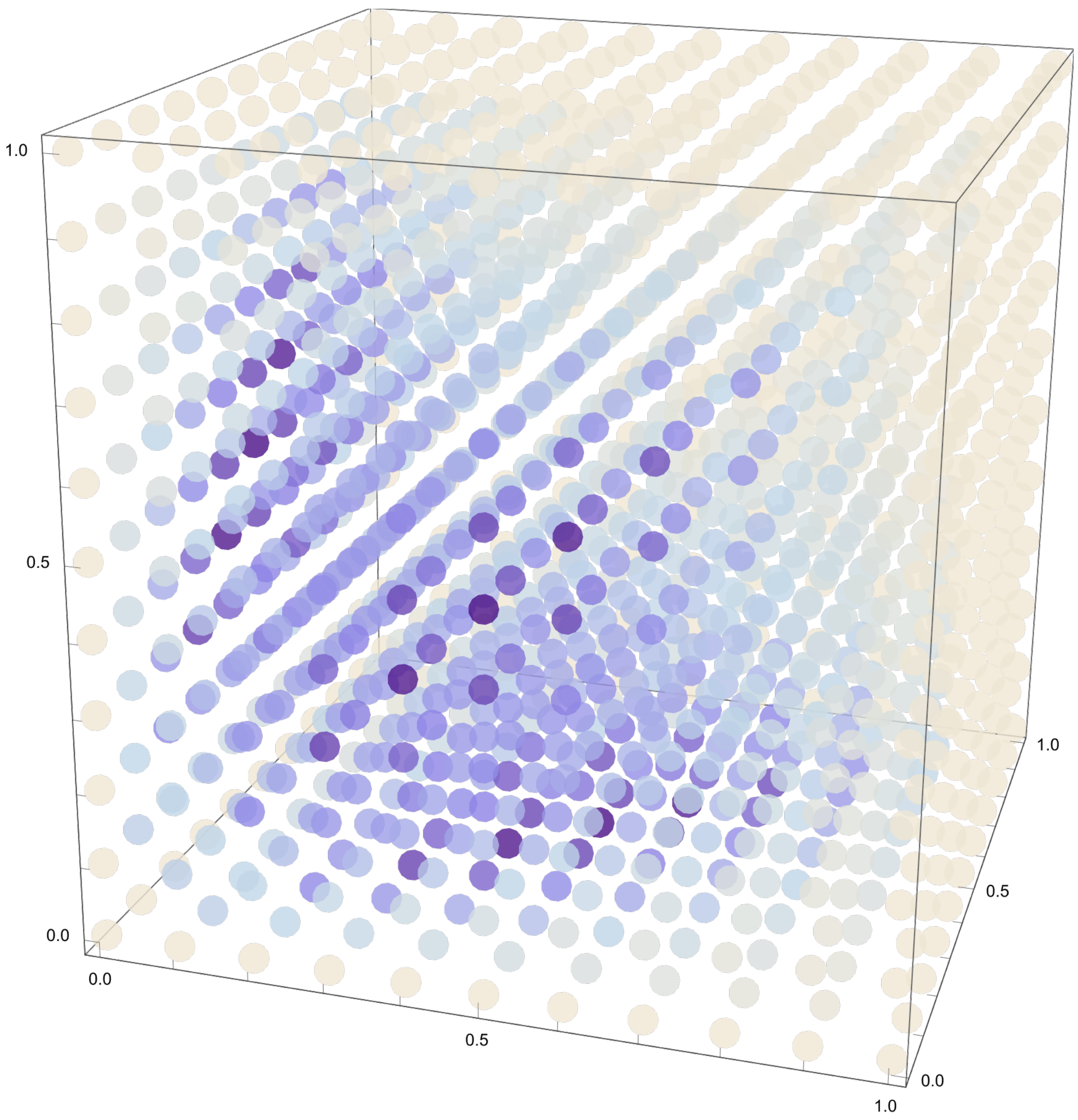

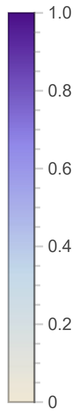


```
(* start with same rates *)
```

```
rates = ConstantArray[10, 8];
(* k1 is gaussian (100,5) *)
k1 = RandomVariate[NormalDistribution[100, 5]]
k2 = RandomVariate[NormalDistribution[100, 5]]
k3 = RandomVariate[NormalDistribution[100, 5]]
k4 = RandomVariate[NormalDistribution[100, 5]]
k5 = RandomVariate[NormalDistribution[100, 5]]
k6 = RandomVariate[NormalDistribution[100, 5]]
k7 = RandomVariate[NormalDistribution[100, 5]]
k8 = RandomVariate[NormalDistribution[100, 5]]


(* Define all diff eqs *)
eqns = {
   x0'[t] == -1 * x0[t] *
     (k1 * y0[t] * z0[t] + k2 * y0[t] * z1[t] + k3 * y1[t] * z0[t] + k4 * y1[t] * z1[t]),
   x1'[t] == -1 * x1[t] *
     (k5 * y0[t] * z0[t] + k6 * y0[t] * z1[t] + k7 * y1[t] * z0[t] + k8 * y1[t] * z1[t]),
   y0'[t] == -1 * y0[t] *
     (k1 * x0[t] * z0[t] + k2 * x0[t] * z1[t] + k5 * x1[t] * z0[t] + k6 * x1[t] * z1[t]),
   y1'[t] == -1 * y1[t] *
     (k3 * x0[t] * z0[t] + k4 * x0[t] * z1[t] + k7 * x1[t] * z0[t] + k8 * x1[t] * z1[t]),
   z0'[t] == -1 * z0[t] *
     (k1 * x0[t] * y0[t] + k3 * x0[t] * y1[t] + k5 * x1[t] * y0[t] + k7 * x1[t] * y1[t]),
   z1'[t] == -1 * z1[t] *
     (k2 * x0[t] * y0[t] + k4 * x0[t] * y1[t] + k6 * x1[t] * y0[t] + k8 * x1[t] * y1[t]),
   f0'[t] ==
    k1 * x0[t] * y0[t] * z0[t] + k4 * x0[t] * y1[t] * z1[t] +
     k6 * x1[t] * y0[t] * z1[t] + k7 * x1[t] * y1[t] * z0[t],
   f0[0] == 0,
   f1'[t] ==
    k2 * x0[t] * y0[t] * z1[t] + k3 * x0[t] * y1[t] * z0[t] +
     k5 * x1[t] * y0[t] * z0[t] + k8 * x1[t] * y1[t] * z1[t],
   f1[0] == 0
  };
(* Define output functions *)
funcs = {f0, f1};
(* Define time range to integrate over *)
trange = {t, 0, 100};

(* Solve the fexp matrix *)
For[i = 1, i < m + 1, i++,
 For[j = 1, j < m + 1, j++,
  For[k = 1, k < m + 1, k++,
   x = range[[i]];
   y = range[[j]];
   z = range[[k]];
```

```
   (* for each case find the new initial conditions *)
   conds = {
     x0[0] == 100 * (1 - x),
     x1[0] == 100 * (x),
     y0[0] == 100 * (1 - y),
     y1[0] == 100 * (y),
     z0[0] == 100 * (1 - z),
     z1[0] == 100 * (z)
    };
   s = NDSolveValue[Join[eqns, conds], funcs, trange];
   (* find f0 and f1 and compute f *)
   a = s[[1]][100]; b = s[[2]][100];
   fexp[[i, j, k]] = b / (b + a);
  ]
 ]
]
(* compute the difference error for each cell *)
For[i = 1, i < m + 1, i++,
 For[j = 1, j < m + 1, j++,
  For[k = 1, k < m + 1, k++,
   fmatrix[[i, j, k]] = Abs[fexp[[i, j, k]] - fpre[[i, j, k]]]
  ]
 ]
]
(* flatten to find the largest error *)
check = Flatten[fmatrix];
max = Max[check]
(* list of form {x,y,z,error(x,y,z)} to plot *)
data = {};
For[i = 1, i < m + 1, i++,
 For[j = 1, j < m + 1, j++,
  For[k = 1, k < m + 1, k++,
   x = range[[i]];
   y = range[[j]];
   z = range[[k]];
   data = Insert[data, {x, y, z, fmatrix[[i, j, k]] / max}, -1];
  ]
 ]
]
mycolor = ColorData[{"BeachColors", "Reverse"}];
Graphics3D[{Opacity[0.8], PointSize[0.03],
  Point[data[[All, 1 ;; 3]] , VertexColors → mycolor /@ data[[All, 4]] ]},
 Axes → True, BoxRatios → {1, 1, 1}, ImageSize → Large]
BarLegend[{"SunsetColors", "Reverse"}, LegendLabel → None]
```

92.5717

98.1011

100.403

98.9604

92.5757

103.226

89.726

101.228

0.0165637

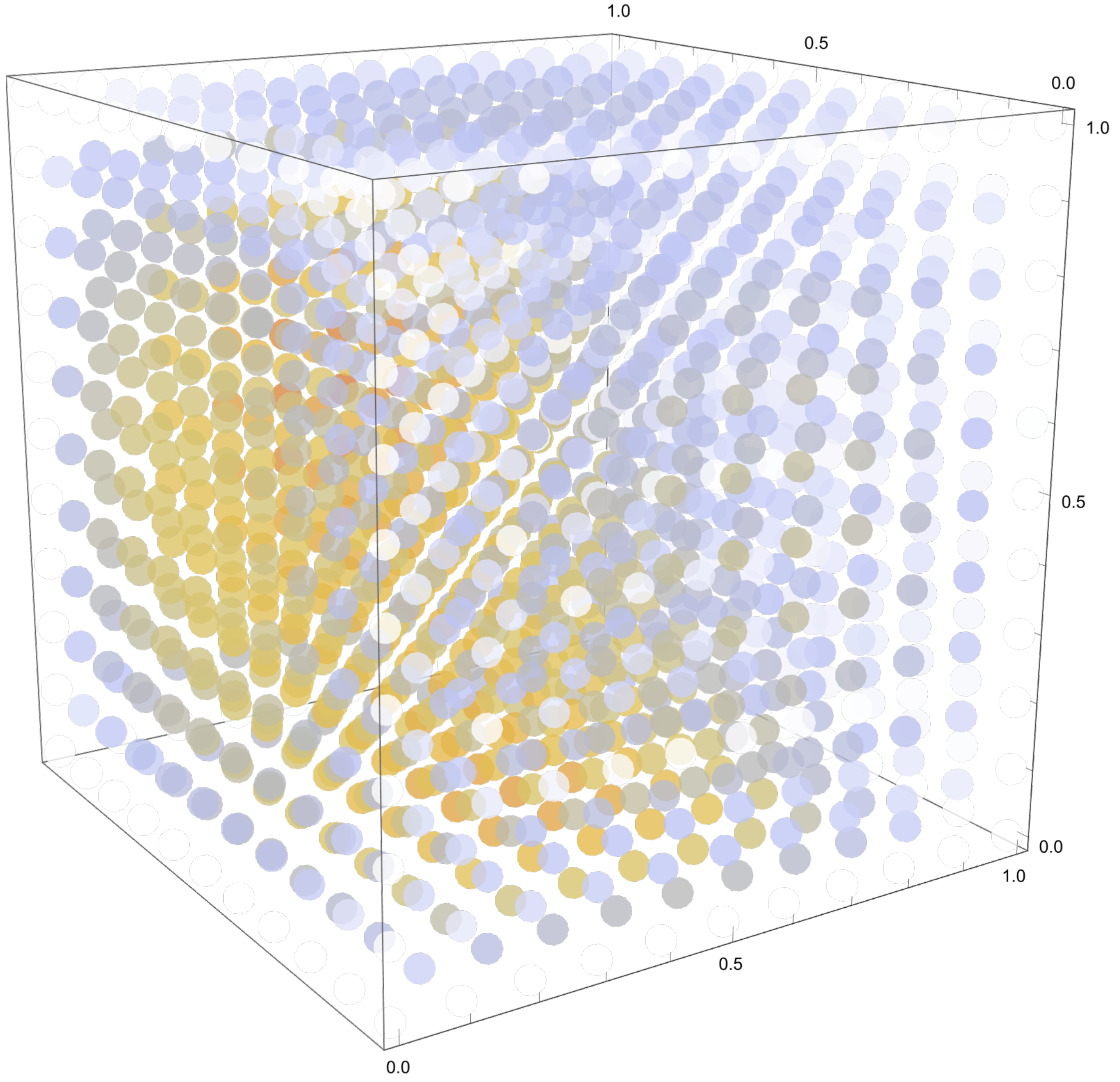

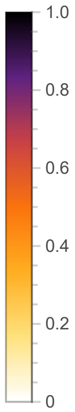
1.0
0.8
0.6
0.4
0.2
0
