# Supplementary material for: Computing mathematical functions with chemical reactions via stochastic logic
Source: PLoS One. 2023 May 8;18(5):e0281574. doi: 10.1371/journal.pone.0281574 (PMC10166555; doi:10.1371/journal.pone.0281574)
Supplement: Attachment — Submitted filename: Response to Reviewers.pdf [file pone.0281574.s003.pdf]

Response to Reviewers

Paper: PONE-D-22-24554
Title: "Computing Mathematical Functions with Chemical Reactions via Stochastic Logic"

We thank the reviewers for detailed, thoughtful reviews. We appreciate that you found the paper well written and easy to follow. We have submitted a revised version incorporating all your suggestions and corrections. (All major changes have been highlighted in red in the revised manuscript.) Below is a summary of the points that you raised, our response, and where specifically in the text we addressed the concern.

Points raised

1) [Reviewer 1] Move the AND gate proof example (Section 5.1) to before the general case or at least advertise that the example will come so the reader who likes examples can read it first.

   We have made this change at lines 243–245 in the revised version.

2) [Reviewer 1] What happens if multiple strands encoding different numbers are simultaneously present in solution? Why not use monomers since multiple molecules could lead to unpredictable results? If I understood the design well, this would lead to unpredictable results. So then, it might be just as well to use a statistical mix of monomers to encode a number.

   A mixture of A_0 and A_1 molecules encodes a stochastic number. So we are, in fact, using a statistical mix of monomers. Please see Section 4.1 for our discussion on this "fractional encoding." Having multiple strands encoding different numbers is not an issue since these strands are "orthogonal" to each other – they do not react with each other. They only react to molecules used for computation (i.e. G_1 from Figure 4). These are designed with specific DNA regions that react with the corresponding monomers. Also note that the stochastic value is calculated using *relative* concentration, so mixing multiple solutions does not change the stochastic value of the variables that these concentration values represent.

3) [Reviewer 1] The authors do not announce the DNA implementation earlier in the paper.

   We now preview the DNA implementation in the introduction, lines 86–89. We also point to it in the abstract.

4) [Reviewer 2] How long are the bitstreams?

   When computing with digital circuits, the length of the bitstream dictates the precision of the computation. The length of the bitstream can be chosen by the user based on their specifications. The more precision that they require, the longer the bitstream that they should use.

In our DNA implementation, the concentration of DNA concatemers corresponds to the length of the bitstreams for the stochastic functions. So the limitation is experimental: how precisely the user can set and measure the input and output concentrations, respectively.

We now discuss this point at lines 501-507.

5) [Reviewer 2] What are the different design specifications for the DNA molecules (length, types of molecules)?

We provide estimates for the lengths of the toeholds and domains at lines 403–408, and discuss how the different molecules corresponding to stochastic variables are constructed using sticky ends (toeholds) and unique sequences (domains) at lines 436–440. We illustrate monomeric design for concatemers, as well as the special sequence for the "fuel" complex G_1, in Figure 4.

6) [Reviewer 2] How are the large number of Boolean variables and rows obtained from the polynomial expansion dealt with?

We note that the number of reactions in a CRN that we design equals the number of rows in the truth table of the corresponding function. The number of rows in a truth table is, of course, exponential in the number of variables: with n variables there are 2^n rows. So, in principle, the approach that we suggest here could lead to CRNs with an unmanageable number of reactions. However stochastic logic permits a wide range of complex functions to be implemented with very simple logic. In the Supplementary Information S1, we provide CRNs for computing polynomial approximations for functions such as arctan, exponential, Bessel, and sinc. All of these are computed by truth tables with 4, 5 or 6 variables. In the field of molecular computing, there is essentially no precedent for computing functions as complex as these. We also note that the structure of our CRNs is uniform and ``feed-forward'': the output species are computed directly from the input species, with no coupling or complex feedback dynamics. Accordingly, the computation should be highly accurate and robust.

We now discuss these points in lines 215–228.

7) [Reviewer 2] How can the output of one CRN be fed into another CRN?

A significant feature of our design is that the encoding of the outputs is the same as that of the inputs. The output of each CRN is encoded by a pair of molecular species, say C_0 and C_1, whose relative concentration encodes a stochastic value, $c = C_1/(C_0 + C_1)$. This is exactly the same format as the inputs, say $a = A_1/(A_0 + A_1)$, and $b = B_1/(B_0 + B_1)$. Therefore the output from a CRN can be used as the input to another CRN. The volume of all input solutions can be scaled up to allow the production of more output solution. This allows for "fanout": dividing the output solution into multiple parts each used as inputs for other CRNs.

We now discuss these points in lines 229–234.

8) [Reviewer 2] How to deal with fanout?

The volume of all input solutions can be scaled up to allow the production of more output solution. This allows for ``fanout'': dividing the output solution into multiple parts each used as inputs to other CRNs. For example, if the output of a CRN feeds into four subsequent CRNs, its inputs must be scaled up by a factor of 4. Its output can then be volumetrically split into four separate units that can be fed into each of the subsequent CRNs.

We now discuss these points in lines 235–240.

9) [Reviewer 2] The multimolecular reactions in the last paragraph of Section 7 are unclear.

The key point is that the "fuel" molecule G_1 can be designed to interact with single strands released from multiple input molecules, not just A or B. So, in this case, a new input D is assumed to react as well. To allow computation using D, the new G_1 molecule would have the sequence $T_1A_iT_2B_jT_4D_lT_3C_kT_3$.

We now updated the corresponding paragraph (lines 493–499) to explain this point better.

10) [Reviewer 2] The error in Section 6.1 should be defined outside of the caption of Figure 2.

We have done this at lines 334–335.

11) [Reviewer 2] Assuming all reactions have the same reaction rate k is an ideal assumption that does not hold in practice with real CRNs. There is no proof that the CRN is robust to reaction rate variations, only a single example.

Our assumption that all reaction rates are equal is a theoretical simplification. We agree that real CRNs will never have this property. However, all our CRNs are uniform, with each reaction having the same number of reactants. The lengths of all DNA toeholds and all domains are identical so the reactions can be expected to exhibit *nearly* equal reaction rates.

We provide Section 6 to mitigate this concern. We deliberately chose the 3-input XOR as our error case because the truth table for XOR is balanced in terms of the number of 0's and 1's. Accordingly, it is the most sensitive to random variations in reaction rates. In contrast, for unbalanced functions, such as AND or OR, errors can readily be masked: computing more 0's for AND or more 1's for OR may not show up statistically. We discuss this point at lines 313–317. Unfortunately, a theoretical proof of robustness to reaction rate variations seems complex and is beyond the scope of the paper.

12) [Reviewer 2] It is not clear how the simulations were performed (e.g. on page 7, how is the computed output 0.462 instead of the expected value of 0.42?)

For each data point (x,y,z), the expected value was calculated using Equation 57. For our simulations, we initialized the input molecule concentrations based on their stochastic values, and then simulated the chemical rate equations for all possible combinations of molecules (X_0,X_1) x (Y_0,Y_1) x (Z_0,Z_1). An example of computing rate equations is shown in Example 5.1. The chemical reactions for the XOR-3 example are given in Equations 58. Here each row of the x y z truth table forms a single equation in a system of 8 differential equations. This system was simulated for a sufficient time window to obtain the convergent values for F_0 and F_1.

Please see Section 6, lines 355–363 for a discussion of this point. We have added our Mathematica script for the error analysis to the Supporting Information.

13) [Reviewer 2] In Equation (16), what kind of operation is x in a x b? Is it just a multiplication between real numbers? Rather, I would have said that if we simulate the CRN we obtain a value c that corresponds to a multiplied by b.

Indeed, the value of c (as it is calculated from C_0 and C_1) corresponds to a times b. It is the multiplication of two real numbers in the [0,1] interval.

We have added line 220 to clarify this point.

Minor corrections (here line numbers in black refer to original submission):

1) line 125: all the outputs -all the inputs
   Done

2) line 231: Eq.20 applies for all t0 -please rephrase this; Eq 20 contains a limit so this does not make sense, better to introduce a new equation here
   Done; added a new equation 21.

3) line 272: than -that
   Done

4) line 284: f -if
   Done

5) lines 409-410: "or the reactant concentration and can easily be broken up" : I could not parse this sentence
   We rewrote that sentence as “and can then be broken up using artificial restriction enzymes”. See lines 450–451* of the revised manuscript.

6) Equations (35), (37), and (44): Explicitly say what the three dots at the beginning of the equations mean (+1 to each eq due to new equation 21)

Replaced all instances of the 3 dots with “therefore”.

7) line 292: We compare -We compared
Done

8) line 337: In Section 7.1 -This is Section 7.1!
Corrected to 7.2

9) line 340: Nucleotide -A nucleotide
Done

10) line 355: stages -stage
Done

11) line 375: molecules -molecule
Done

12) page 18, line 4 of caption of Figure 3: reactant -reactants
Done

13) Ref.[8]: still to appear?
Fixed

14) Ref.[12]: venue is missing
Fixed

15) Ref.[46]: some problems in the title
Fixed
